# Adiponectin Signaling Pathways in Liver Diseases

**DOI:** 10.3390/biomedicines6020052

**Published:** 2018-05-07

**Authors:** Tania Gamberi, Francesca Magherini, Alessandra Modesti, Tania Fiaschi

**Affiliations:** Dipartimento di Scienze Biomediche, Sperimentali e Cliniche “Mario Serio”, Università degli Studi di Firenze, Viale Morgagni 50, 50134 Firenze, Italy; tania.gamberi@unifi.it (T.G.); francesca.magherini@unifi.it (F.M.); alessandra.modesti@unifi.it (A.M.)

**Keywords:** adiponectin, liver, signaling pathway

## Abstract

In the liver, adiponectin regulates both glucose and lipid metabolism and exerts an insulin-sensitizing effect. The binding of adiponectin with its specific receptors induces the activation of a proper signaling cascade that becomes altered in liver pathologies. This review describes the different signaling pathways in healthy and diseased hepatocytes, also highlighting the beneficial role of adiponectin in autophagy activation and hepatic regeneration.

## 1. Adiponectin in Healthy Liver

### 1.1. Introduction

Adiponectin is one of the several hormones secreted by adipose tissue [1]. Besides the latter, several other tissues are autocrine for adiponectin production, such as bone [2,3], placenta [4], cardiomyocytes [5], pituitary gland [6], and skeletal muscle [7], thus generating a local high concentration of the hormone. Adiponectin is secreted as a monomer called “full-length” adiponectin (fAd) which associates to form complexes with different molecular weights circulating in the plasma [8]. In the local microenvironment, fAd can be cleaved in the “globular form” adiponectin (gAd) by the elastase secreted by activated monocytes, neutrophils [9], and macrophages [10], thus suggesting that the conversion of fAd into gAd could be driven by local inflammation. In plasma, adiponectin circulates as three complexes called low molecular form (LMW, composed of fAd trimer), middle molecular form (MMW, composed of fAd hexamer), and high molecular form (HMW, composed of the association of 12–18 fAd). Distinct downstream biological effects depend on the complex distribution in the plasma [11]. For example, males have lower levels of HMW with respect to females, accounting for the different total levels of adiponectin. HMW complex induces a more marked decreased of glucose following intraperitoneal HMW injection in mice [11]. Correct adiponectin folding and assembly is key step in the regulation of plasma levels and complex distribution. The assembly and secretion of HMW requires the hydroxylation and glycosylation of four lysines on adiponectin. Mutations on these sites greatly decreased HMW concentration in plasma, thus leading to decreased biological effects [12]. Moreover, hexamers of adiponectin require the formation of the inter-trimeric disulfide bond at cysteine 39 (cysteine 36 in human adiponectin). Mutation of this cysteine firstly abolishes hexamer formation and then HMW complexes [13]. Adiponectin binds at two specific receptors, AdipoR1 and AdipoR2, which belong to the seven-transmembrane domain receptor family but are not coupled to G protein. The two receptors are differently expressed among tissues, since AdipoR1 is ubiquitously produced, while AdipoR2 is predominantly expressed in the liver [14].

Hepatic stellate cells (HSC) and Kupffer cells constitutively express the same amount of AdipoR1 and AdipoR2 [15,16]. HSC are activated following a trauma and lead to the secretion of collagen and to the formation of scar tissue, leading to chronic fibrosis or cirrhosis. Kupffer cells are key mediators of both liver injury and repair.

Binding experiments showed that fAd and gAd have different affinities for AdipoR1 and AdipoR2. Indeed, fAd has higher affinity for AdipoR2, while gAd preferably interacts with AdipoR1 [17]. In addition to AdipoR1 and AdipoR2, T-cadherin, a glycosylphosphatidylinositol-anchored protein, is the receptor for the hexameric and high molecular weight complexes of adiponectin [18]. To date, many studies have shown that T-cadherin exerts its effects in healthy and diseased vascular endothelium [19,20].

### 1.2. Signaling Pathway of Adiponectin in Healthy Liver

In healthy liver, adiponectin controls the metabolism of both glucose and lipids, decreasing gluconeogenesis and stimulating glycolysis and fatty acid oxidation. These metabolic effects occur through the binding of gAd to AdipoR1 and fAd to the specific hepatic receptor AdipoR2. Following the binding of the ligand to the receptor, the adaptor protein phosphotyrosine interaction, pH domain, and leucine zipper containing 1 (APPL1) associate to AdipoR1 or AdipoR2. This binding involves the phosphotyrosine domain of APPL1 and the N-terminal domain of the receptor [21] and requires the formation of the homodimer APPL1:APPL1 [22]. The two receptors trigger two distinct signaling pathways, since AdipoR1 promotes AMP-activated kinase (AMPK) activation while AdipoR2 stimulates peroxisome proliferator-activated receptor α (PPARα) cascade. AMPK is partially activated by the binding to AMP and becomes totally activated following phosphorylation on threonine 172 by the serine/threonine protein liver kinase B1 (LKB1). Activated AMPK promotes the inhibition of phosphoenolpyruvate carboxykinase (PEPCK) and glucose 6-phosphatase (G6Pase) transcription, thus leading to the decrease of gluconeogenesis [23]. In addition, AMPK blocks lipid synthesis via the inhibitory phosphorylation of Acetyl-CoA carboxylase (ACC) that catalyzes the synthesis of malonyl-CoA. Malonyl-CoA is both the precursor of fatty acid biosynthesis and a potent inhibitor of carnitine palmitoyl transferase I (CPT-I), the enzyme that controls the transfer into the mitochondria of the long-chain fatty Acyl-CoA for fatty acid oxidation. Therefore, adiponectin-AMPK signaling promotes lipid catabolism and opposes triglyceride formation in the liver [24]. Activated AMPK phosphorylates Ser372 of the sterol regulatory element binding protein 1c (SREBP-1c), leading to the repression of SREBP-1c, a master regulator of fatty acid synthesis [25,26]. In the liver, free fatty acid burning is due also to the signaling cascade triggered by PPARα, which cooperates with AMPK in promoting fatty acid combustion [14].

Although it was shown that AdipoR1 and AdipoR2 regulate glucose and fatty acid metabolism via the activation of AMPK and PPARα pathways, additional signaling molecules participate at the pleiotropic actions of adiponectin in the liver. A recent paper describing the crystal structure of AdipoR2 bound to a free fatty acid molecule revealed an intrinsic basal ceramidase activity of the receptor that is enhanced by adiponectin binding [27]. The involvement of adiponectin in the decrease of intracellular level of ceramide has previously been reported. Adiponectin, through its receptors AdipoR1 and AdipoR2, induces ceramidase activation and thus leads to decreased levels of hepatic ceramide and promotes ceramide catabolism with the formation of sphingosine-1 phosphate in an AMPK-independent mechanism [28]. This peculiar function of adiponectin has been further confirmed in mice overexpressing AdipoR1 and AdipoR2 in the liver. Increased levels of adiponectin receptors lead to reduced ceramide levels and the amelioration of total body glucose and lipid homeostasis in an adiponectin-dependent mechanism [29]. High intracellular levels of ceramide greatly compromise insulin signaling, thus predisposing the liver to the onset of insulin resistance and the development of type II diabetes [30]. In agreement, obese individuals with lowered glucose tolerance and insulin sensitivity show enhanced ceramide levels and high ceramide synthase expression in visceral white adipose tissue [31]. In mice, the deletion of ceramide synthase leads to decreased ceramide and improved glucose tolerance and insulin sensitivity under high-fat diet challenge [31]. The beneficial effects of adiponectin, specifically its insulin-sensitizing effect, are probably closely associated to the ceramidase activity of its own receptors.

**Figure 1:**
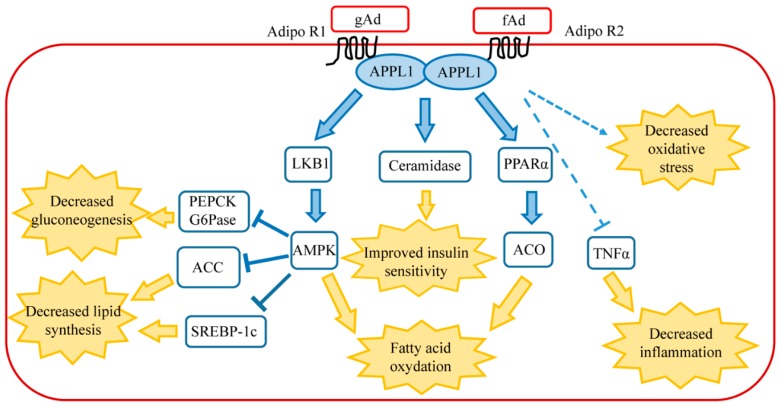
Main signaling pathways of adiponectin in healthy liver. AdipoR1 activates AMPK pathway, whereas AdipoR2 activates peroxisome proliferator-activated receptor α (PPARα). Connections between AdipoR1/AdipoR2 signaling, TNFα, ceramide, and oxidative stress are described in the text. The final biological effects of each signaling pathway are shown following the yellow arrows. Abbreviations: ACC, Acetyl-CoA carboxylase; ACO, Acyl CoA oxidase; AdipoR1, Adiponectin receptor 1; AdipoR2, Adiponectin receptor 2; AMPK, AMP-activated protein kinase; APPL1, adaptor protein phosphotyrosine interaction, pH domain, and leucine zipper containing 1; fAd, full-length adiponectin; gAd, globular adiponectin; G6Pase, glucose 6-phosphatase; LKB1, liver kinase B1; PEPCK, phosphoenolpyruvate carboxykinase; PPARα, peroxisome proliferator-activated receptor α; SCREB-P1c, sterol regulatory element binding protein 1c; TNFα, tumor necrosis factor α.

Animal-based studies have demonstrated that adiponectin possesses potent protective activities against various forms of liver injuries [32]. Although the mechanism is not yet clear, some evidences suggest that adiponectin directly opposes the damaging effects of TNFα within the liver tissue [33]. In animal models of both alcoholic and non-alcoholic steatohepatitis, exogenous adiponectin reduces hepatomegaly, depletes lipid accumulation, quenches hepatic inflammation, and decreases hepatic expression and plasma concentrations of TNFα. In fact, inflammatory cytokines are key mediators of hepatic inflammation, cell death, and fibrosis, as well as regeneration after massive or small liver injury [32]. In particular, TNFα alters lipid metabolism by interfering with several crucial transcriptional regulators, such as SREBP-1c [34]. It has been further demonstrated that TNFα provokes the processing of SREBP-1c in ethanol-exposed hepatoma cell lines, resulting in the inappropriate induction of lipogenic enzymes, thus suggesting that adiponectin may counteract hepatic lipid accumulation through the antagonism of TNFα [35]. Adiponectin levels are negatively associated with mediators of inflammation, including interleukin-6 (IL-6) and C-reactive protein (CRP), and positively related to the anti-inflammatory cytokine IL-10 [36]. In the liver, cytokines such as IL-6 and TNFα are mainly produced from Kupffer cells and HSC, and partly from inflamed hepatocytes [32,37]. In agreement with the anti-inflammatory role of adiponectin, recent studies have demonstrated that hypoadiponectinemia is associated with inflammasome activation. Inflammasome is a multiprotein complex that plays a crucial role in the proteolytic activation by caspases of the cytokines belonging to the interleukin family [38]. It has been reported that hypoadiponectinemia promotes the activation of the inflammasome that is responsible for vascular endothelium dysfunction. This event occurs through a mechanism involving the formation of oxidative and nitrative stress [39]. Moreover, gAd inhibits interleukin-1β (IL-1β) production following lipopolysaccharide stimulation in macrophages. In these cells, gAd suppresses the activation of inflammasome through autophagy induction and AMPK signaling activation [40]. The main signaling cascades triggered by adiponectin in the liver are shown in Figure 1.

### 1.3. Reactive Oxygen Species (ROS) in Adiponectin Signaling in Healthy Liver

Adiponectin signaling leads to the formation of ROS, which act as second messengers both in the liver and in skeletal muscle [41]. Particularly in hepatocytes, the binding of gAd to receptors leads to a transient generation of ROS involving the activation of the small GTPase Rac1 and 5-lipoxygenase (5-LOX). This intracellular burst of ROS induces a ligand-independent transactivation of Insulin Receptor (IR) through the oxidation and inactivation of PTP1B, a phosphotyrosine phosphatase controlling IR phosphorylation. Downstream effects of adiponectin in hepatocytes, such as increased glycogen synthesis, aerobic consumption of glucose, and the activation of the Mitogen Activated Protein Kinase (MAPK) cascade, are ROS-dependent. Adiponectin signaling involving ROS formation could partly explain the mechanism through which adiponectin exerts in vivo insulin-mimetic effects [42].

If, on one hand, adiponectin promotes ROS production to be used as second messengers, on the other adiponectin counteracts the excessive accumulation of oxygen products, thus impeding the generation of intracellular oxidative stress. It has been reported that oxidative stress is harmful for adiponectin action, i.e., in adipose tissue, adiponectin secretion is inhibited by oxidative stress [43,44]. In hepatocytes, the binding of adiponectin to its cognate receptors leads to the activation of aldehyde oxidase-1 (AOX-1), which reduces intracellular levels of ROS through the increased activation of PPARα [45].

## 2. Adiponectin in Liver Diseases

A protective action of adiponectin in liver injury is now emerging from several clinical and animal studies. Recent studies have evaluated the relationship between adiponectin levels and some liver diseases, such as non-alcoholic fatty liver disease (NAFLD), non-alcoholic steatohepatitis (NASH), hepatic fibrosis, and hepatocellular carcinoma (HCC). The findings suggest that hypoadiponectinemia in obese people may be an important risk factor for the clinical progression of these chronic liver diseases. NAFLD, which is the most common liver disease affecting about 20–30% of the general population in Western countries, encompasses a wide range of liver injuries ranging from simple steatosis, NASH, to progressive fibrosis. Furthermore, NASH may lead to liver cirrhosis and/or HCC [46]. The onset of liver disease is caused by the deregulated production of many cytokines by adipocyte tissue. In particular, visceral fat accumulation and an inflamed condition enhances the serum levels of such cytokines, predisposing tissue to the onset of hepatic diseases (as TNF-α, resistin, and leptin) and the concomitant downregulation of protective cytokines (such as adiponectin) [47]. The key molecules in adiponectin signaling that are altered in these hepatic diseases are described below and summarized in Table 1.

### 2.1. Alcoholic Liver Disease (ALD)

ALD is a consequence of excessive alcohol consumption and could be considered as the first step in the progression towards more severe forms of liver injury, including steatohepatitis, fibrosis, and cirrhosis [48]. The disease is characterized by the increased accumulation of fat in the liver and several studies have abundantly suggested a main role of adiponectin in the onset of alcoholic fatty liver. Firstly, ethanol inhibits adiponectin expression and secretion both in cultured adipocytes and in chronically ethanol-fed animals, thus generating a low level of circulating adiponectin. Moreover, chronic ethanol consumption in mice leads to the selective downregulation of AdipoR2 in the liver [49]. Although the precise mechanism leading to AdipoR2 downregulation is unknown, the involvement of the axis sirtuin1 (Sirt1)-forkhead box O1 (FoxO1) is postulated. Indeed, FoxO1 positively regulates AdipoR1/R2 expression [50] while Sirt1 could promote FoxO1 nuclear localization, increasing the transcriptional activity [51]. Chronic ethanol exposure impairs lipid metabolism pathways triggered by key transcriptional regulators, such as AMPK, peroxisome proliferator-activated receptor gamma coactivator alpha (PGC-1α), PPARα, and SREBP-1, leading to the excessive accumulation of fat in the liver. Forcing the increase of adiponectin leads to a great amelioration of the pathology. For example, the administration of fAd to ethanol-fed mice corrected alterations in AMPK signaling and prevented hepatic steatosis [52]. Moreover, increased circulating adiponectin levels induced by resveratrol were associated with enhanced hepatic expression levels of Sirt1 and reduced hepatic lipid accumulation in chronically ethanol-fed mice [49]. The dysregulation of TNFα is greatly involved in the pathogenesis of alcoholic liver injury [53] and adiponectin directly opposes the damaging effects of TNFα [33]. TNFα alters lipid metabolism by interfering with several crucial transcriptional regulators such as AMPK and SREBP-1 [34,54]. It has been demonstrated that TNFα leads to the induction of lipogenic enzymes in an SREBP-1-dependent manner in ethanol-exposed hepatoma cell lines [35]. Furthermore, gAd attenuates the production of TNFα and ROS in Kupffer cells exposed to ethanol [55]. Thus, adiponectin protects the liver against alcoholic injury through its anti-inflammatory activity and counteracts hepatic liver accumulation through the antagonism of TNFα. It has been recently reported that adiponectin levels are increased in patients with cirrhosis. In this case, increased levels of adiponectin are associated with hepatic injury and worse prognosis in patients with alcoholic liver disease [56].

### 2.2. Non-Alcoholic Fatty Liver Disease (NAFLD)

NAFLD is characterized by insulin resistance (IR) and is commonly associated with obesity and type II diabetes (T2DM). Although the pathogenesis of NAFLD is not fully elucidated, oxidative stress, along with inflammation, plays important roles in the development and progression of the disease. However, the main risk factor for NAFLD is considered visceral adiposity, which is associated with an elevated circulating level of free fatty acids (FFA) and adipocyte hypertrophy. In addition, macrophage infiltration of adipose tissue activates pro-inflammatory cytokines (such as TNFα, IL-6, IL-1β) and nitric oxide [57]. These alterations can lead to the reduced transcription of adiponectin mRNA and adiponectin secretion by adipocytes. Adiponectin and TNFα are mutually inhibited when IL-6 suppresses the expression of adiponectin [58]. Several clinical studies have upheld a role for a low circulating adiponectin level in the pathogenesis of NAFLD and confirmed the strong association among reduced adiponectin production by adipose tissue, NAFLD, and IR, together with the hypothesis that an imbalance between pro-inflammatory and anti-inflammatory cytokines may have a pathogenic role in the development of liver damage in NAFLD [58].

### 2.3. Non-Alcoholic Steatohepatitis (NASH)

Even though obesity and/or insulin resistance are hallmarks of NASH, the specific basis of the pathology remains to be clarified. It is certain that the progression of fatty liver (steatosis) into NASH is associated to a chronic inflammatory state, triggered by oxidative stress along with lipid peroxidation [36]. Indeed, steatosis is considered the first step in the onset of liver fibrosis, while the second step is represented by mitochondrial damage that leads to ROS production followed by peroxidation, the release of inflammatory cytokines, the death of hepatocytes, and the activation of HSC [23].

Studies using animal models straightened the correlation among hypoadiponectinemia, steatosis, and NASH. Feeding mice with the choline-deficient l-amino acid-defined diet, Kamada et al. demonstrated that hepatic steatosis was more severe in adiponectin knockout (KO) mice than in wild-type mice, while the overexpression of adiponectin resulted in the attenuation of hepatic steatosis. Adiponectin KO mice showed enhanced expression of two rate-limiting enzymes in fatty acid synthesis, namely ACC and fatty acid synthase, thus lacking the protective effect of adiponectin against fat accumulation in the liver [47]. Considering adiponectin receptors, Matsunami et al. examined the roles of hepatic AdipoR1/R2 and Insulin Receptor Substrate-1/-2 (IRS-1/-2) in the onset of NASH [59]. Rats fed a high-fat and high-cholesterol diet for eight weeks showed the hallmarks of NASH, namely fatty liver with inflammation and fibrosis. In this condition, the expression levels of AdipoR1/R2 significantly decreased, whereas IRS-1 was significantly increased. Results demonstrated that AdipoR1/R2 and IRS might be crucially important regulators for the synthesis and oxidation of fatty acids in the liver with NASH. However, the correlation between adiponectin receptors and NASH remains unclear, as contradictory results have been published. In fact, AdipoR1/R2 expressions has been reported to be both significantly decreased [60] and increased [61] in the onset of NASH. The beneficial effects of adiponectin leading to the amelioration of NASH and liver fibrosis occurs by inhibition of Kupffer cells and HSC activation that are responsible for the secretion of anti-inflammatory cytokines [62]. The attenuation of pro-inflammatory cytokine production due to adiponectin in these cells is partially mediated by attenuating the translocation of NFκB to the nucleus [63], also occurring through the adiponectin-dependent expression of the anti-inflammation cytokine interleukin-1-receptor antagonist (IL-1RA) [64]. Wang and colleagues found that adiponectin KO mice showed an increased lipid accumulation even under normal feeding, suggesting a possible correlation between this pre-existing hepatic steatotic condition and dysregulated mitochondria functions. In particular, the beneficial effects of adiponectin on mitochondria respiratory chain activities involved uncoupling protein 2 (UCP2), a mitochondria inner membrane transporter. The level of UCP2 is decreased in the liver of adiponectin KO mice and is significantly upregulated by adiponectin treatment [65]. The effects of adiponectin on mitochondria respiratory chain activities are dramatically attenuated in UCP2-deficient mice, thus suggesting that the increased UCP2 expression is mandatory for eliciting beneficial adiponectin effects. A growing body of evidence suggests that UCP2 may play a beneficial role in various stages of fatty liver diseases. UCP2 possesses anti-oxidant activities through the inhibition of ROS production from mitochondria and can inhibit the production of pro-inflammatory cytokines in both macrophage and Kupffer cells [66,67,68]. Although these results suggest the existence of a reciprocal relationship between uncoupling proteins and adiponectin, the detailed signaling mechanisms underlying adiponectin-induced UCP2 expression are not yet clear and warrant further investigation [46].

As far as clinical studies are concerned, serum adiponectin levels have been found to be inversely related to the presence of NASH [58]. In fact, plasma adiponectin level is decreased in patients with steatosis and NASH, correlating with the severity of liver histology. Hypoadiponectinemia is a characteristic feature of patients with NASH, but the cause for the further decrease of systemic adiponectin levels during transition from fatty liver to NASH is still unclear. Patients with NASH display damaged mitochondria and impaired oxidative phosphorylation (OxPhos) complexes [44,45,46]. The resulting dysfunctional mitochondrial respiratory chain triggers increased ROS level followed by lipid peroxidation, which in turn causes steatohepatitis, necrosis, inflammation, and fibrosis. Moreover, ROS production in steatohepatitis could directly injure mitochondrial DNA (mtDNA) and OxPhos complexes, as well as induce NFκB activation and the hepatic synthesis of TNFα [44].

### 2.4. Hepatic Fibrosis

The transformation of HSC into myofibroblasts is the key step initiating the fibrotic process during liver injury [69]. Activated HSCs, which constitutively express both AdipoR1 and AdipoR2 [70], induce the amount of the extracellular matrix. Experimental evidences have demonstrated that adiponectin inhibits hepatic fibrosis through the AMPK pathway, acting on multiple molecular mechanisms responsible for the onset of pathology. Indeed, this adipokine can maintain HSC quiescence as well as avoid their migration properties by means of the inhibition of the platelet-derived growth factor (PDGF) stimulation [53,54]. In addition, adiponectin can also inhibit HSC activation through the downregulation of the transforming growth factor beta 1 (TGF-β1) expression [55]. In this context, Tomita K et al. reported a relationship between TGF-β1 and AdipoR2 expression levels, demonstrating that the AdipoR2 silencing leads to increased TGF-β1 mRNA levels. Conversely, AdipoR2 overexpression reduces TGF-β1 levels [71]. In agreement, AdipoR2 KO mice fed with a methionine-choline deficient diet, which causes progressive fibrosis steatohepatitis, exhibited higher levels of steatosis, inflammation, and fibrosis [47]. Leptin is a well described pro-fibrotic adipokine and several studies have shown that adiponectin antagonizes leptin activity [47]. Adiponectin blocks leptin-induced STAT3 phosphorylation in activated HSC and leptin-mediated upregulation of tissue inhibitor of matrix metalloproteinases-1 (TIMP-1) release both in vitro and in vivo [57,72].

Fatty liver represents an important step towards the onset of liver fibrosis [73], the development of which is greatly affected by oxidative stress. Aldehyde oxidase 1 (AOX1) activity, which has been identified as an important source of ROS, is reduced in hepatocytes by adiponectin via the activation of PPARα signaling [45]. Adiponectin also increases ROS detoxifying enzymes and is involved in the induction of superoxide dismutase 1 and catalase through AdipoR2 [74].

### 2.5. Hepatocellular Carcinoma (HCC)

Abusive alcohol consumption and NAFLD are important risk factors for the development of cirrhosis and hepatocellular carcinoma (HCC) [58,75]. Recent studies showed that plasma adiponectin levels are inversely correlated with the risk of cancers [47,76]. In clinical studies, hypoadiponectinemia is correlated with colorectal cancer [77], gastric cancer [78], prostate cancer [79], endometrial cancer [80], and breast cancer [81]. In addition, in vitro studies have revealed that adiponectin regulates cell proliferation in cell lines originating from various types of cancer, including prostate cancer [82,83], HCC [84], breast cancer [85,86], leukemia [87], and esophageal cancer [88]. Several preclinical studies have also confirmed the involvement of adiponectin in liver tumor formation. Experiments performed in choline-deficient mice showed that adiponectin depletion induces liver cirrhosis, thus promoting the onset of hepatic tumor.

Saxena et al. demonstrated that adiponectin inhibits HCC cell proliferation and displays a pro-apoptotic effect. In HCC, adiponectin stimulation activates caspase-3 and increases the phosphorylation of c-Jun N-terminal kinase (JNK), leading to apoptosis cell death [84]. Moreover, microarray analysis performed on human HCC pointed out an inverse correlation between adiponectin level and tumor size [84]. Overall, the experimental evidences might suggest anti-tumor properties of adiponectin. For example, Al-Gayyar et al. highlighted the chemoprotective and hepatoprotective functions of adiponectin by means of the inhibition of sulfatase-2 activities [89]. Nevertheless, the precise role of adiponectin in the onset of HCC remains to be clarified.

## 3. Adiponectin in Liver Regeneration and Autophagy

### 3.1. Adiponectin and Liver Regeneration

Beside the anti-fibrotic and anti-inflammatory effects described in the previous section, adiponectin also displays regenerative properties in the liver. Liver is one of a few adult organs that can regenerate after hepatic resection or other injuries. Ezaki et al. investigated liver regeneration after partial hepatectomy in adiponectin KO mice. In particular, they demonstrated that the regeneration process is delayed during the first 72 h in adiponectin KO mice in comparison to wild-type mice. In agreement, the expression of cyclins D1, A2, and B1 is lower in KO than in wild-type mice. Furthermore, hepatic triglyceride content was increased 72 h after partial hepatectomy in KO mice in comparison to wild-type mice. The increased triglyceride level is due to impaired fatty acid oxidation correlated with the low expression level of CPT-I and PPARα mRNAs [90]. Shu et al. obtained similar results using a different mouse strain [91]. In particular, they observed decreased cell proliferation and increased hepatic level of triglycerides and cholesterol. These data suggest that the downstream events mediated by adiponectin receptors are required for the progression of liver regeneration. Interestingly, these events seem to not include AMPK signaling, since the phosphorylation/activation of this enzyme remains unchanged during liver regeneration.

Partial hepatectomy is known to induce the activation of different signaling pathways. In particular, the activation of the JAK-STAT3 pathway by IL-6 plays a key role in liver regeneration [92]. Shu et al. demonstrated that in adiponectin KO mice, STAT3 phosphorylation is reduced during the first 24–72 h after partial hepatectomy, while the level of Socs3, a protein blocking JAK-STAT3 pathway, is increased [91], thus highlighting a prominent role of adiponectin in liver regeneration.

### 3.2. Adiponectin and Autophagy in Damaged Liver

Several clinical studies have proved that a low level of circulating adiponectin is an independent risk factor for diabetes, hypertension, atherosclerosis, and NASH [93,94,95]. On the other hand, elevating plasma levels of adiponectin, induced by genetic or pharmacological approaches, alleviates these disorders [52,96]. Furthermore, adiponectin ameliorates other forms of liver diseases, including alcoholic fatty liver disease and steatohepatitis [52], carbon tetrachloride and bile duct ligation-induced cirrhosis [97], and lipopolysaccharide/d-galactosamine-induced liver injury [98]. These protective effects of adiponectin are mediated by the direct involvement of the hormone in several processes, including the promotion of mitochondrial fatty acid oxidation in hepatocytes [52], the suppression of HSC activation [99], and the inhibition of pro-inflammatory cytokine production in Kupffer cells [62]. Although the precise mechanisms whereby adiponectin exerts its hepatoprotective effects have not yet been elucidated, adiponectin-induced autophagy could play a predominant role. Indeed, autophagy is activated as a cellular adaptive mechanism to prevent cell damage in response to liver injury. The activation of this process ensures the removal of damaged cell structures as mitochondria, thus protecting against mitochondria damage-induced oxidative stress and necrotic cells [100]. An association between adiponectin and autophagy activation in several cell populations has been reported [101,102,103,104]. Under some circumstances, autophagy constitutes a stress adaptation that avoids cell death by suppressing apoptosis, whereas in other cellular conditions, it constitutes an alternative cell-death pathway [105]. Concerning hepatic cells, adiponectin induces the activation of autophagy to protect hepatocytes from apoptosis induced by ethanol [106,107]. The anti-apoptotic effect elicited by adiponectin occurs through the suppression of the ethanol-induced activation of caspase-8 and Bax expression. AMPK plays an important role in the induction of autophagy. Indeed, AMPK silencing blocked the adiponectin-induced expression of autophagic genes, which in turn prevented protection from ethanol-induced apoptosis.

In addition, Lin et al. reported that adiponectin-induced autophagy plays an essential role in maintaining the health of hepatic cells [108]. The authors showed that acetaminophen (APAP) treatment leads to adiponectin accumulation in damaged liver and that adiponectin deficiency renders mice more susceptible to APAP overdose-induced hepatotoxicity and mortality. These negative effects are reverted by exogenous adiponectin replenishment. In particular, adiponectin counteracts APAP negative effects by the activation of autophagy, thus leading to the removal of damaged mitochondria and the amelioration of oxidative stress and necrosis. Dissecting the molecular basis, the authors showed that the activation of autophagy by adiponectin is mediated by AMPK in primary hepatocytes. In particular, it has been shown that AMPK activation is associated with the increased phosphorylation of serine 317 and the decreased phosphorylation of inhibitory serine 757 of Unk51-like kinase 1 (ULK1) mediated by the autophagic inhibitor mTOR [109]. This finding suggests that adiponectin could promote autophagy activation indirectly via mTOR suppression. Notably, the beneficial effects due to adiponectin-induced autophagy, namely the amelioration of APAP-induced mitochondria dysfunction and hepatic necrosis, are completely abrogated by the genetic or pharmacological inhibition of AMPK, suggesting that AMPK is a mandatory upstream activator of adiponectin-induced autophagy in primary hepatocytes [108].

It is very interesting to note that the activation of autophagy is able to ameliorate several other liver diseases, including alcoholic and non-alcoholic fatty liver diseases, liver fibrosis, and viral hepatitis [110,111]. Notably, these diseases can be mitigated by the therapeutic administration of adiponectin [47,112]. Therefore, the activation of autophagic processes could represent a common mechanism whereby adiponectin exerts a protective effect against various forms of acute and chronic liver injury.

## 4. Concluding Remarks

Over the last years, adiponectin has emerged as an important regulator for the development of several hepatic diseases. In particular, hypoadiponectinemia could be considered a key factor promoting the development of NAFLD, NASH, and hepatic tumor formation. Indeed, several animal models of hepatic syndromes show a slight correlation between the onset of hepatic disease and reduced circulating adiponectin levels, decreased expression of adiponectin receptors, and impaired adiponectin-mediated signaling. Base on this, adiponectin could be considered as a biomarker of those hepatic pathologies, such as NAFLD, NASH, and hepatic tumor, that have been shown to be associated with hypoadiponectinemia. On the other hand, an increase of adiponectin levels induces a marked improvement of many liver diseases, thus demonstrating, once again, the prominent role of adiponectin in the protection against the onset of the hepatic syndromes. Based on the beneficial effects induced by adiponectin, future efforts must be directed towards increasing adiponectin and its hepatic receptor levels by pharmacological strategies, involving, for example, treatment with PPARγ agonists. Moreover, future studies should indicate whether the positive effects induced by increased levels of adiponectin could be achieved by supplying adiponectin exogenously. Finally, a deeper study would be useful for understanding the role in the onset of hepatic diseases of the individual multimeric forms of adiponectin and their possible use in the amelioration/cure of these pathologies.

## Figures and Tables

**Table 1:** The main effects of adiponectin in cells or signalling molecules in different liver diseases are shown. The arrows indicate adiponectin-induced increase (up arrow) or decrease (down arrow). Abbreviations: ACC, Acetyl-CoA carboxylase; AMPK, AMPK-activated protein kinase; AOX-1, aldehyde oxidase-1; CPT I, Carnitine palmitoyl-transferase I; HCC, hepatocarcinoma cells; HSC, hepatic stellate cells; IL-1β, interleukin 1-beta; IL-6, interleukin-6; IL-1RA, anti-inflammation cytokine interleukin-1-receptor antagonist; JNK, c-Jun N-terminal kinase; PGC-1α, peroxisome proliferator-activated receptor gamma coactivator alpha; PPARα, peroxisome proliferator-activated receptor alpha; ROS, Reactive Oxygen Species; SIRT1, sirtuin1; SREBP-1, sterol regulatory element-binding protein; STAT3, Signal Transducer and Activator of Transcription 3; TIMP-1, Tissue Inhibitor of Matrix metalloproteinases-1; TGFβ, Tumour Necrosis Factor β; TNFα, Tumour Necrosis Factor α.

	ALD	NASH	Hepatic Fibrosis	Hepatocellular Carcinoma	NAFLD
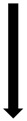	- mRNA adiponectin -adiponectin secretion -AdipoR2 -CPT I activity -AMPK activity -SIRT-1 activity -PGC-1α/PPARα activity	-plasma adiponectin -AdipoR1 -AdipoR2 -NFkB nuclear migration -activation of Kupffer and HSC cells	-AOX-1 activity -phospho-STAT3 -monocyte chem. protein 1 -TGFβ1 -HSC quiescence -HSC proliferation -HSC migration	-sulfatase-2 activity	-mRNA and secretion of adiponectin
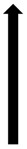	-activation of Kupffer cells -TNF-α level -IL-1β level -IL-6 level -SREBP-1 activity -ACC activity -acid synthase activity	-acid synthase level -ACC level -IL-1RA	-TIMP-1 -ROS detoxifying enzymes -superoxide dismutase -catalase	-apoptosis of HCC -caspase-3 -phospho-JNK	
